# Occurrence of two myxosporean parasites in the gall bladder of white seabream *Diplodus sargus* (L.) (Teleostei, Sparidae), with the morphological and molecular description of *Ceratomyxa sargus* n. sp.

**DOI:** 10.7717/peerj.14599

**Published:** 2023-01-13

**Authors:** Sónia Rocha, Luís Filipe Rangel, Graça Casal, Ricardo Severino, Florbela Soares, Pedro Rodrigues, Maria João Santos

**Affiliations:** 1 Institute of Biomedical Sciences Abel Salazar (ICBAS), University of Porto, Porto, Portugal; 2 Instituto de Investigação e Inovação em Saúde (i3S), University of Porto, Porto, Portugal; 3 Laboratory of Animal Pathology, Interdisciplinary Centre of Marine and Environmental Research (CIIMAR), University of Porto, Porto, Portugal; 4 Department of Biology, Faculty of Sciences (FCUP), University of Porto, Porto, Portugal; 5 TOXRUN – Toxicology Research Unit, University Institute of Health Sciences, CESPU, CRL, Gandra, Portugal; 6 IPMA, Aquaculture Research Station, Olhão, Portugal

**Keywords:** Phylogeny, SSU rRNA gene, Aquaculture, Ultrastructure, Taxonomy, Ceratomyxa sargus n. sp., Zschokkella auratis, Myxosporean parasites

## Abstract

Myxosporeans are widespread cnidarian parasites that usually parasitize fish as part of their complex life cycle, thus constituting a potential threat for the aquaculture industry. White seabream *Diplodus sargus* (L.) is a commercially valuable sparid fish reared in Southern European aquacultures. Nonetheless, knowledge on myxosporean infections potentially harming the sustainable production of this fish is extremely limited. In this study, a myxosporean survey was conducted on *D. sargus* specimens reared in two Southern Portuguese fish farms. Two coelozoic myxosporeans were detected infecting the gall bladder, and are herein reported based on microscopic and molecular procedures: *Ceratomyxa sargus* n. sp. and *Zschokkella auratis* Rocha et al., 2013, previously described from reared stocks of gilthead seabream *Sparus aurata* in the same geographic locality. *Ceratomyxa sargus* n. sp. is the 12^th^ species of the genus to be reported from Southern European sparids, reinforcing a substantial radiation of *Ceratomyxa* within this fish family and geographic region. SSU rRNA-based Bayesian inference and maximum likelihood analyses revealed *C. sargus* n. sp. positioned separately from other sparid-infecting *Ceratomyxa* spp. reported from Southern European countries, demonstrating that this species does not share a more immediate common ancestor with its closest relatives based on host affinity and geography. The recognition of a novel sparid-infecting lineage within the *Ceratomyxa* clade strengthens the contention that this genus entered sparid fish multiple times, namely in the Southern European region. The identification of *Zschokkella auratis* infections in *D. sargus* demonstrates that host shift has occurred among sparids reared in the Southern Portuguese coast. This agrees with the broad host specificity that is usually attributed to this genus, and that may be suggested to be the outcome of the capacity of the *Zschokkella* morphotype to undergo host shift/switch based on our findings and the limited molecular data available for this genus. Thus, a better understanding of *Zschokkella* host-associated diversification and dispersal mechanisms requires the increasing availability of molecular data from infections of the same species occurring in multiple hosts and geographical locations.

## Introduction

Myxosporeans are obligate cnidarian parasites with a complex life cycle that usually involves fish as vertebrate hosts. They constitute a diverse and widespread group with more than 2,400 species described (Okamura, Gruhl & Bartholomew, 2015). *Ceratomyxa* Thélohan, 1892 is the second most speciose myxosporean genus, comprising near 300 species that represent ca. 13% of the taxa currently known. This genus is typically coelozoic, parasitizing the gall bladder of marine teleosts, and exceptionally occurring in elasmobranchs (Eiras, 2006; Lom & Dyková, 2006; Eiras, Cruz & Saraiva, 2018). In turn, the genus *Zschokkella* Auerbach, 1909 comprises ca. 97 species, most of which are coelozoic and, less frequently, histozoic in freshwater and marine fishes worldwide, including three representatives described from amphibians and reptiles (Lom & Dyková, 2006; Matsche et al., 2021). Several studies have shown that species identification within these genera based on myxospore morphology is artificial (Kent et al., 2001; Holzer, Sommerville & Wootten, 2004; Fiala, 2006; Bartošová, Fiala & Hypša, 2009). However, the lack of a specified threshold for myxosporean interspecific variability makes the usage of morphological features a requirement for differentiating between species (Atkinson et al., 2015; Gunter, Whipps & Adlard, 2009; Bartošová-Sojková et al., 2018). As such, reports of both novel and known species are presently based on the comprehensive analysis of several characters that include myxospore morphology, sequencing of selected molecular markers (usually the SSU rRNA gene), host species, tissue tropism, and geographic locality (Atkinson et al., 2015).

Despite the relative abundance of *Ceratomyxa* sequences presently available in GenBank, the interrelationships and evolutionary drivers of these parasites remain unknown, as geography and host affinity do not appear to correlate with relatedness of species within the *Ceratomyxa* clade (Gunter & Adlard, 2008; Heiniger, Gunter & Adlard, 2008; Gunter, Whipps & Adlard, 2009; Fiala et al., 2015; Rocha et al., 2015; Bartošová-Sojková et al., 2018; Alama-Bermejo et al., 2021). In turn, the molecular data currently available for *Zschokkella* is relatively scarce, with phylogenetic analyses retrieving the genus as polyphyletic (Fiala, 2006; Rocha et al., 2013; Fiala et al., 2015). The progressive expansion of the data available on genetic databases is therefore a key objective of myxosporean research, helping unequivocal species identification, but also the recognition of evolutionary patterns.

Several sparid fish are commercially important for Southern European aquaculture industries. Cultured stocks mainly comprise the gilthead seabream *Sparus aurata* L., but also sharpsnout seabream *Diplodus puntazzo* (Walbaum, 1792), white seabream *Diplodus sargus* (L.), common dentex *Dentex dentex* (L.), and blackspot seabream *Pagellus bogaraveo* (Brünnich, 1768). Parasitological studies have shown that the sustainable production of sparids in this geographical region is threatened by myxosporean infections, several of which caused by *Ceratomyxa* spp. (Georgévitch, 1916; Lubat et al., 1989; Diamant, Lom & Dyková, 1994; Sitjà-Bobadilla, Palenzuela & Álvarez-Pellitero, 1995; Sitjà-Bobadilla & Alvarez-Pellitero, 1995, 2001; Palenzuela, Sitjà-Bobadilla & Alvarez-Pellitero, 1997; Athanassopoulou, Prapas & Rodger, 1999; Kalavati & MacKenzie, 1999; Caffara et al., 2003; Diamant et al., 2005; Golomazou et al., 2009; Alama-Bermejo, Raga & Holzer, 2011; Rocha et al., 2013, 2015; Rangel et al., 2014; Thabet et al., 2019). Myxosporean surveys conducted on Portuguese fish farms have mostly focused on *S. aurata* and European seabass *Dicentrarchus labrax* (L.), which are known to host species belonging to the genera *Ceratomyxa*, *Kudoa* Meglitsch, 1947, *Ortholinea* Shulman, 1962, *Zschokkella* Auerbach, 1909 and *Sphaerospora* Thélohan, 1892 in this geographic location (Santos, 1996; Costa et al., 1998; Rocha et al., 2013, 2015, 2016; Rangel et al., 2014, 2016, 2017). Despite these fishes being commonly reared together with *D. sargus* in semi-intensive polyculture regimes established in the Portuguese Southern coast, myxosporean surveys have not been performed in specimens of *D. sargus*. Worldwide, a single study by Golomazou et al. (2006) reported the occurrence of myxosporean infections in commercial stocks of this fish, including a *Myxobolus* sp. in the kidney, a *Kudoa* sp. in the musculature, and *Enteromyxum leei* in the intestine of specimens reared in cages off Greece. Another study by Sirin, Santos & Rangel (2018) further described the myxosporean *Bipteria lusitanica* from wild specimens captured off the North Atlantic Portuguese coast.

Considering the above, this study aimed to provide knowledge regarding the diversity of myxosporean parasites occurring in reared populations of *D. sargus*. To pursue this objective, specimens were sampled from two Southern Portuguese fish farms and their external and internal tissues examined for the presence of myxosporean infection. Herein, we describe the occurrence of two myxosporeans in the gall bladder of *D. sargus* based on myxospore morphology, sequencing of the SSU rRNA gene, and host and tissue data.

## Materials and Methods

### Fish sampling and myxosporean survey

Specimens of reared white seabream *Diplodus sargus* (L.) (Teleostei, Sparidae) were collected from earth ponds located in the Algarve Atlantic coast in southern Portugal. Fifty-four specimens were collected between April 2014 and March 2015 from a fish farm (37°08′N/08°37′W) near the city of Portimão, with further 48 specimens collected between March 2021 and March 2022 from the IPMA’s Aquaculture Research Station (EPPO—Estação Piloto de Piscicultura de Olhão) (37°01′N 7°49′W), near the city of Olhão. All the people involved in animal handling and experimentation received proper training (category B courses accredited by FELASA, the Federation of Laboratory Animal Science Associations) and all fish facilities were accredited by the Portuguese National Authority for Animal Health (DGAV) with the Number 0421/2018. All experimental procedures involving animals followed the European Directive 2010/63/EU and the related guidelines and Portuguese legislation (Decreto-Lei 113/2013) for animal experimentation and welfare. Freshly caught specimens were dissected for performing a myxosporean survey of several organs and tissues, including brain, eye, muscle, gills, heart, spleen, liver, gall bladder, gonads, swim bladder, urinary bladder, kidney, and digestive tube. Fresh pseudoplasmodia and myxospores were observed and photographed using an Olympus CX23 light microscope (Olympus, Tokyo, Japan), equipped with the Olympus EP50 digital camera (Olympus, Japan). Myxospore morphometry was determined following the guidelines of Lom & Arthur (1989). All measurements herein provided were determined from a minimum of 25 myxospores, and include the mean value ± standard deviation, and range of variation.

The electronic version of this article in Portable Document Format (PDF) will represent a published work according to the International Commission on Zoological Nomenclature (ICZN), and hence the new names contained in the electronic version are effectively published under that Code from the electronic edition alone. This published work and the nomenclatural acts it contains have been registered in ZooBank, the online registration system for the ICZN. The ZooBank LSIDs (Life Science Identifiers) can be resolved and the associated information viewed through any standard web browser by appending the LSID to the prefix http://zoobank.org/. The LSID for this publication is: urn:lsid:zoobank.org:pub:E70B1814-0651-4396-81B4-0CFECEC525E2. The online version of this work is archived and available from the following digital repositories: PeerJ, PubMed Central SCIE and CLOCKSS.

### Electron microscopy

Myxospores and pseudoplasmodia obtained from infected gall bladders were fixed in 5% glutaraldehyde buffered in 0.2 M sodium cacodylate (pH 7.4) for 20–24 h at 4 °C, rinsed in buffer, post-fixed in 2% osmium tetroxide in 0.2 M sodium cacodylate buffer (pH 7.4) for 3–4 h, rinsed in buffer, and dehydrated in a graded series of ethanol. For transmission electron microscopy (TEM), samples of the *Ceratomyxa* species were then embedded using ascending mixtures of EPON in oxide propylene, ending in EPON. Semithin sections cut from EPON blocks were stained with methylene blue-Azure II, and ultrathin sections double contrasted with uranyl acetate and lead citrate. Ultrathin sections were then observed and photographed using a JEOL 100 CXII TEM (JEOL Optical, Tokyo, Japan), operated at 60 kV. For scanning electron microscopy (SEM), dehydrated samples of the *Zschokkella* species were critical point dried, coated with a gold-palladium alloy (60%), and observed and photographed with a JSM-6301F SEM (JEOL Optical, Tokyo, Japan), operated at 15 kV.

### DNA extraction, amplification, and sequencing

Myxospores belonging to the *Ceratomyxa* and *Zschokkella* morphotypes were obtained from bile, each from three separate individuals. Samples were fixed and preserved in absolute ethanol at 4 °C. Genomic DNA extraction was performed using a GenElute™ Mammalian Genomic DNA Miniprep Kit (Sigma-Aldrich, St Louis, MI, USA), following the manufacturer’s instructions.

The SSU rRNA gene of both myxosporeans was amplified and sequenced using the universal primers and myxosporean-specific primers shown in Table 1. PCR reactions and cycling conditions were performed as in Rocha et al. (2015). Electrophoresis of the obtained PCR products was performed in a 1% agarose 1× Tris-acetate-EDTA buffer (TAE) gel stained with ethidium bromide. PCR products were purified using the ExoFast method and sequenced directly using a BigDye Terminator v1.1 from the Applied Biosystems kit (Applied Biosystems, Carlsbad, CA, USA), and ABI3700 DNA analyzer (Perkin-Elmer, Waltham, MA, USA; Applied Biosystems, Carlsbad, CA, USA; Stabvida, Oeiras, Portugal).

**Table 1:** Polymerase chain reaction primers used for the amplification and sequencing of the SSU rRNA gene of *Ceratomyxa sargus* n. sp. and *Zschokkella auratis*.

Name	Sequence (5′–3′)	Paired with	Source
18E	CTG GTT GAT CCT GCC AGT	MyxospecR, MYX4R	Hillis & Dixon (1991)
MyxospecF	TTC TGC CCT ATC AAC TTG TTG	MYX4R, 18R	Fiala (2006)
MYX4F	GTT CGT GGA GTG ATC TGT CAG	18R	Hallett & Diamant (2001)
MyxospecR	CAA CAA GTT GAT AGG GCA GAA	18E	Fiala (2006)
MYX4R	CTG ACA GAT CAC TCC ACG AAC	18E, MyxospecF	Hallett & Diamant (2001)
18R	CTA CGG AAA CCT TGT TAC G	MyxospecF, MYX4F	Whipps et al. (2003)

### Sequence assembly, distance estimation, and phylogenetic analysis

Forward and reverse sequence segments of the *Ceratomyxa* and *Zschokkella* isolates were manually aligned with ClustalW in MEGA X software (Nei & Kumar, 2000; Kumar et al., 2018), with ambiguous bases clarified using corresponding ABI chromatograms. For performing distance estimation analysis, a dataset comprising 33 sequences was constructed and included the new *Ceratomyxa* isolate, all highly similar putative *Ceratomyxa* species (above a 90% cut-off) determined by BLASTn, and all sequences available for congeners that infect sparids or that share similar myxospore morphology and geographic location. In turn, the dataset used for distance estimation of the new *Zschokkella* isolate comprised all SSU rRNA sequences available in GenBank for this genus. Datasets were aligned using the software MAFFT version 7 available online, and distance estimation was performed in MEGA X, with the p-distance model selected, and all ambiguous positions removed for each sequence pair.

For the phylogenetic analysis of the new *Ceratomyxa*, the dataset used for distance estimation was broadened to encompass other representative *Ceratomyxa* sequences, *Palliatus indecorus* (DQ377712), *Myxodavisia bulani* (KM273030), *Unicapsulocaudum mugilum* (KP091845), and the outgroup sequences of *Kudoa thyrsites* (AY542482), and *Ellipsomyxa mugilis* (AF411336). Alignments were performed using the software MAFFT version 7 available online. Ambiguous characters were removed using Gblocks v0.91b with less stringent parameters (Castresana, 2000; Dereeper et al., 2008). Phylogenetic trees were calculated using maximum likelihood (ML) and Bayesian inference (BI). ML analyses were conducted in MEGA X using the general time reversible model with gamma distributed rate and invariant sites (GTR + G + I), selected based on the lowest score of Bayesian information criterion (BIC) and corrected Akaike information criterion (AIC), and with bootstrap confidence values calculated from 1,000 replicates. BI analyses were conducted in MrBayes v.3.2.6 (Ronquist & Huelsenbeck, 2003), with the general time reversible model with gamma-shaped rate variations across sites (Invgamma) (GTR + I + G) selected. Posterior probability distributions were generated using the Markov chain Monte Carlo method. Four chains were run simultaneously for 2 million generations, with burn-in set at 25%, and trees sampled every 500 generations.

## Results

Analyzed fish specimens did not present external signs of infection or disease, and neither was morbidity or mortality reported from the sampled fish stocks. The macro- and microscopic analysis of 13 different organs revealed the occurrence of myxosporean infection in the gall bladder by *Ceratomyxa* Thélohan, 1892 and *Zschokkella* Auerbach, 1909, in the kidney by *Sphaerospora* Thélohan, 1892, and in the urinary bladder by *Ortholinea* Shulman, 1962. Co-infection in the gall bladder was determined based on myxospores observation and could be detected in four out of the 54 specimens collected from the fish farm near the city of Portimão. Infections by *Zschokkella* were not observed at the EPPO sampling location. The microscopic and molecular procedures performed identified the ceratomyxid as new species that is herein described as *Ceratomyxa sargus* n. sp., and the second isolate as *Zschokkella auratis* Rocha et al., 2013.

### *Ceratomyxa sargus* n. sp. urn:lsid:zoobank.org:act:3103EB6E-000F-445C-AA10-F129C0509EFC

Taxonomic placement

Phylum Cnidaria Hatschek, 1888

Sub-phylum Myxozoa Grassé, 1970

Class Myxosporea Bütschli, 1881

Order Bivalvulida Shulman, 1959

Family Ceratomyxidae Doflein, 1899

Genus *Ceratomyxa* Thélohan, 1892

**Morphological description and taxonomic summary**

**Figure 1:**
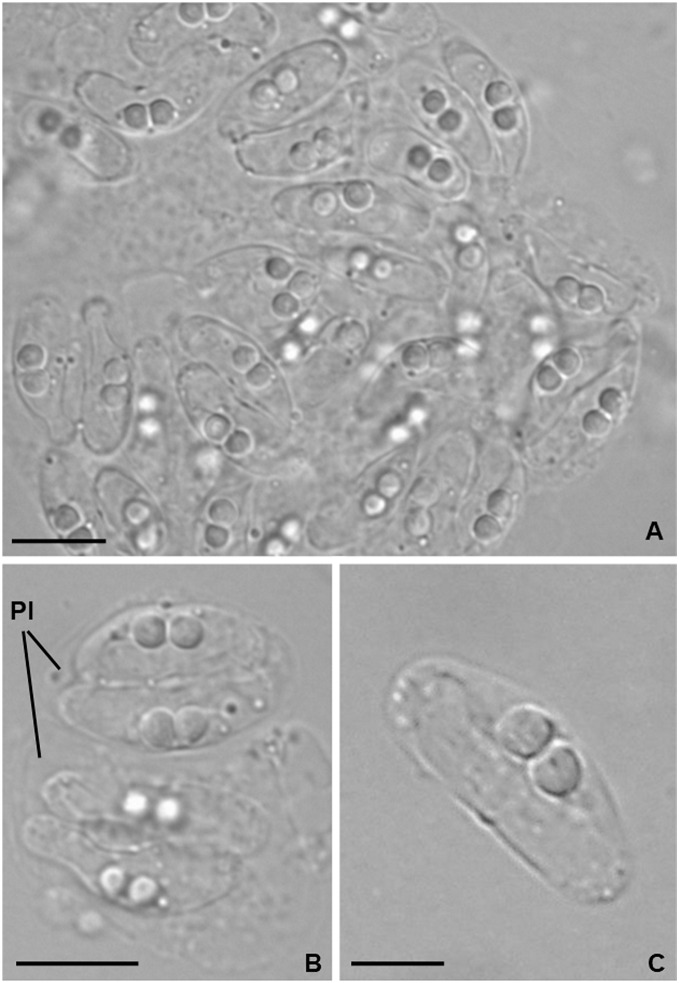
Light micrographs of *Ceratomyxa sargus* n. sp. from the gall bladder of *Diplodus sargus*. (A) Cluster of plasmodia and free myxospores. Scale bar = 10 µm. (B) Disporic pseudoplasmodia (Pl). Scale bar = 10 µm. (C) Free crescent-shaped myxospore. Scale bar = 5 µm.

**Figure 2:**
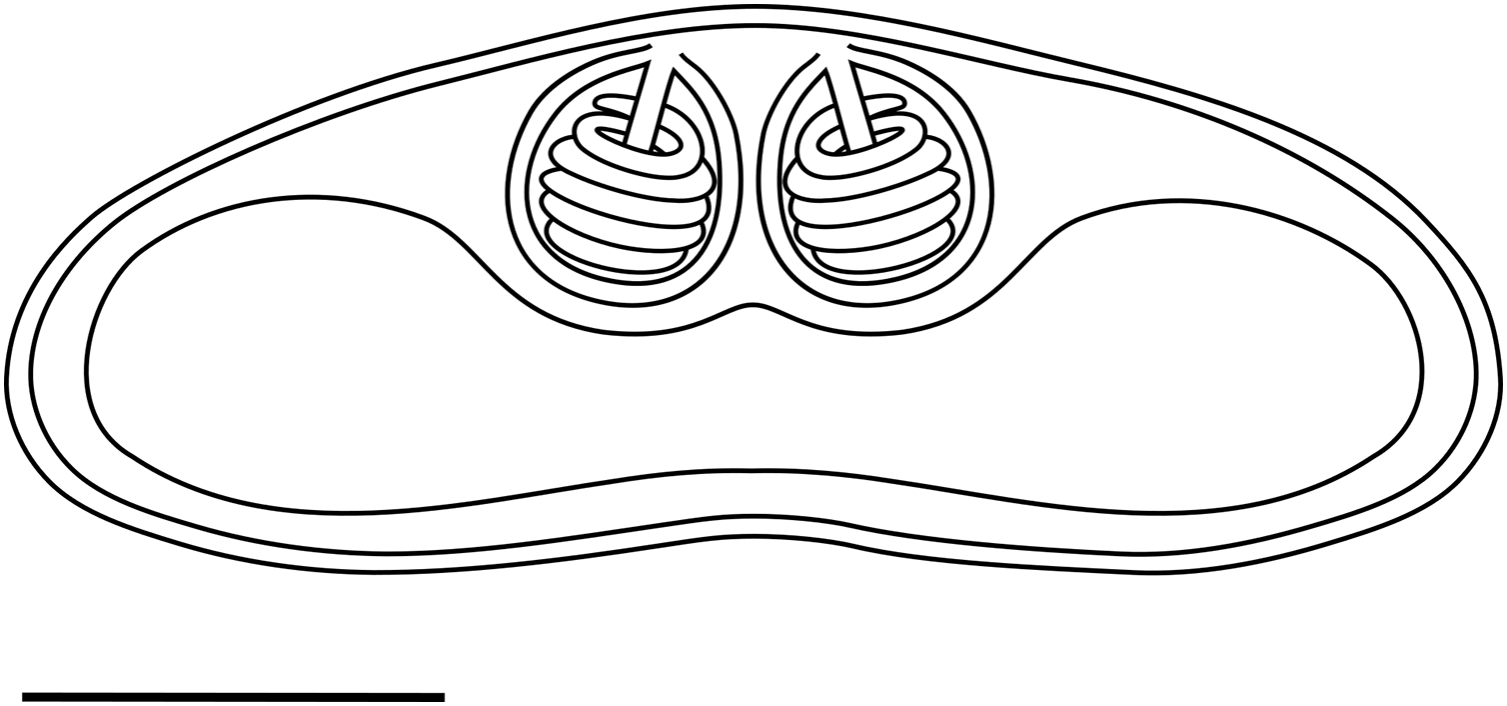
Schematic drawing of a mature myxospore of *Ceratomyxa sargus* n. sp. from the gall bladder of *Diplodus sargus* Scale bar = 5 µm.

*Light microscopy*. Pseudoplasmodia at different stages of maturation, and mature myxospores, observed free in the bile (Fig. 1). Young pseudoplasmodia spherical; mature pseudoplasmodia subspherical to elliptical, mostly disporic (Figs. 1A and 1B). Mature myxospores crescent-shaped with convex anterior margin and slightly concave to straight posterior margin; valves with rounded ends. Myxospores 4.9 ± 0.4 (4.2–5.7) µm long and 14.5 ± 1.1 (12.4–16.9) µm thick. Polar capsules located at the same level at the myxospores anterior pole, equally-sized and subspherical, 2.2 ± 0.2 (1.9–2.5) µm long and 1.9 ± 0.2 (1.5–2.3) µm wide (Fig. 1C). A schematic drawing of a myxospore in valvular view is provided in Fig. 2.

**Figure 3:**
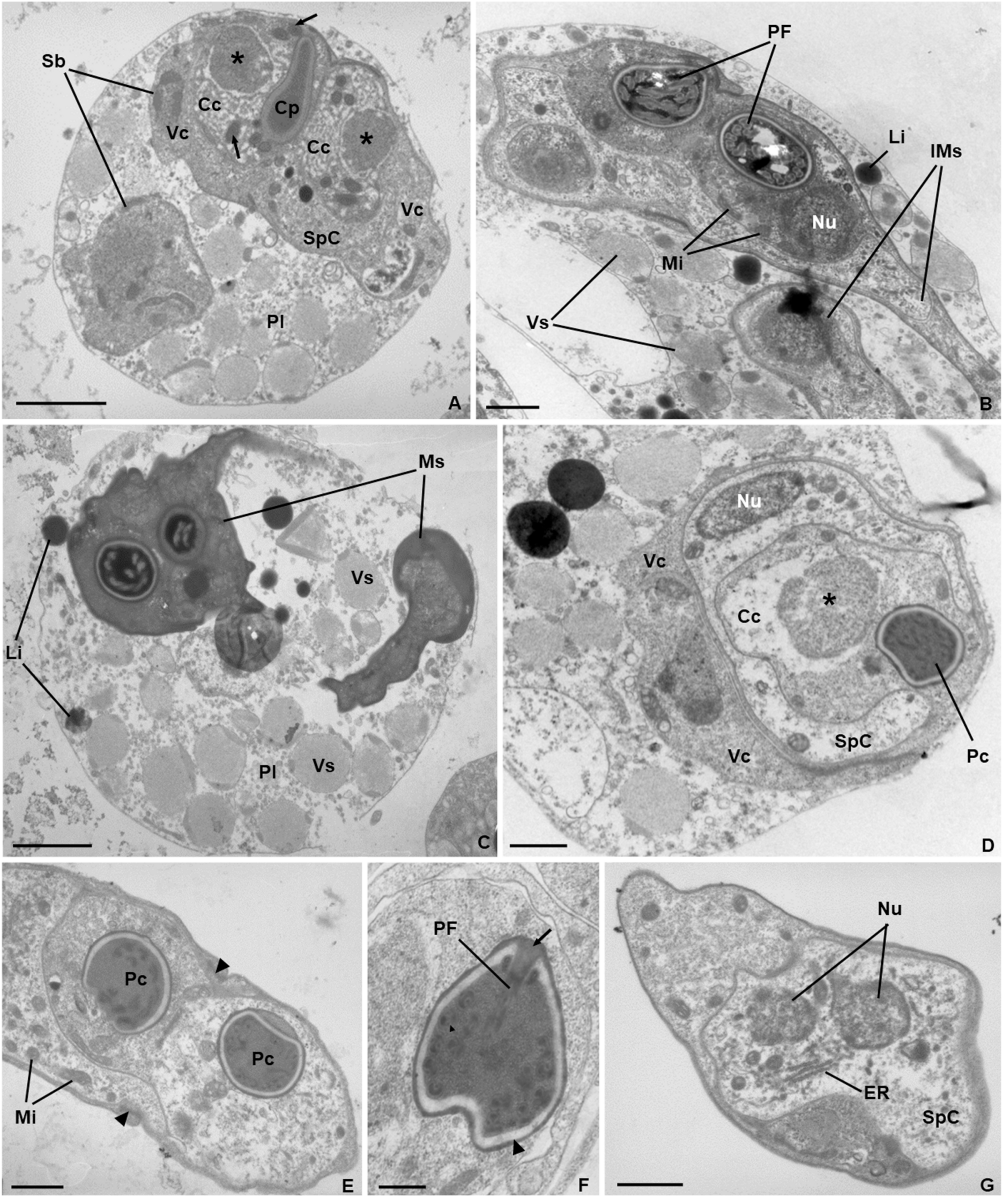
Transmission electron micrographs of *Ceratomyxa sargus* n. sp. from the gall bladder of *Diplodus sargus*. (A) Pseudoplasmodium (Pl) containing two developing sporoblasts (Sb), one of which displaying all sporogenic cells: two valvogenic cells (VC) surrounding two capsulogenic cells (CC) and one sporoplasmogenic cell (SpC). Each capsulogenic cell presents a single nucleus (*), and a globular capsular primordium (Cp) that extends into an external tubule (arrows). Scale bar = 2 µm. (B) Pseudoplasmodium containing two immature myxospores (IMs), numerous vesicles (Vs), and some lipidic globules (Li). Notice that the polar capsules are completely formed, with the polar tubule coiling within, but the cytoplasmic content of the capsulogenic cells remains, inclusive the nuclei (Nu) and several mitochondria (Mi). Scale bar = 1 µm. (C) Pseudoplasmodium (Pl) containing two fully matured myxospores (Ms), numerous vesicles, and some lipidic globules. Scale bar = 2 µm. (D) Transverse section of an immature myxospore showing its valvogenic cells (VC), one of the two capsulogenic cells (CC) displaying its degrading nucleus (*) and an almost completely matured polar capsule (PC), and the sporoplasmogenic cell (SpC) also displaying one of its two nuclei (Nu). Scale bar = 1 µm. (E) Oblique section of a myxospore showing its almost matured polar capsules (PC) located in the proximity of the curved suture line (arrowheads) that unites the valves (V). The sporoplasm is riddled with mitochondria (Mi). Scale bar = 1 µm. (F) Longitudinal section of a polar capsule displaying the polar tubule (PT) coiled internally to the double-layered wall (arrowhead) and capped at the apex by a stopper (arrow). Scale bar = 0.5 µm. (G) Oblique section of a myxospore, evidencing the cytoplasmic content of the sporoplasmogenic cell (SpC), in which two nuclei (Nu), and endoplasmic reticulum (ER) can be observed. Scale bar = 1 µm.

*Ultrastructure*. Young and mature pseudoplasmodia with smooth surface membrane and displaying numerous vesicles containing granular material of variable electronic density, as well as lipidic droplets, distributed in the cytoplasm (Figs. 3A–3D). More mature pseudoplasmodia containing two sporoblasts in equivalent developmental stages (Figs. 3A–3C). Younger sporoblasts comprising two large and single nucleated valvogenic cells that surround two capsulogenic cells and one sporoplasmogenic cell (Fig. 3A). Capsulogenic cells uninucleate, initially with a globular capsular primordium extending into an external tubule (Fig. 3A). In more advanced stages of sporogenesis, the tubule is coiled within the inner wall of the capsular primordium, forming a young polar capsule and its internal polar tubule (Figs. 3B–3D). Myxospores constituted by two symmetrical valves, thick and smooth, united along a curved suture line (Fig. 3E). Polar capsules with a double-layered wall (outer layer thinner and electron-dense; inner layer thicker and electron-lucent), and a homogenous dense matrix. Polar tubule coiled in two rows, forming ca. five turns in the outer row, and two turns in the inner row (Fig. 3F). Sporoplasm binucleate, displaying endoplasmic reticulum and several mitochondria (Fig. 3G).

*Type host. Diplodus sargus* (L.) (Eupercaria *incertae sedis*, Sparidae) (common name: white seabream).

*Type locality*. The Algarve Atlantic coast in southern Portugal, from a fish farm (37°08′N/08°37′W) near the city of Portimão, and another (37°01′N 7°49′W) near the city of Olhão.

*Site of infection*. Gall bladder.

*Prevalence of infection*. 11.8% (13.0%, 7 infected in 54 specimens analysed from the fish farm near the city of Portimão; 10.0%, 5 infected in 48 specimens analysed from the EPPO).

*Pathogenicity*. Analysed fish did not present external signs of infection or disease.

*Type material*. Series of phototypes of the hapantotype, deposited together with a representative DNA sample in the Natural History and Science Museum of the University of Porto, Portugal, reference CIIMAR 2022.66.

*Molecular data*. Partial SSU rRNA gene sequence with 1,849 bp and GenBank accession number ON123709.

*Etymology*. The specific epithet “*sargus*” refers to the specific epithet of the host species.

**Differential diagnosis**

The myxospores of *Ceratomyxa sargus* n. sp. closely resemble those of *C. bartholomewae* Gunter, Burger & Adlard, 2010, *C. cardinalis* Heiniger & Adlard, 2013, *C. cribbi* Gunter & Adlard, 2008, *C. cyanosomae* Heiniger & Adlard, 2013, *C. dehoopi* Reed et al., 2007, C. *moseri* Gunter & Adlard, 2008, *C. puntazzi* Alama-Bermejo, Raga & Holzer, 2011, *C. rueppelli* Heiniger & Adlard, 2013 and *C. siganicola* Zhang et al., 2019 in terms of shape, but can be readily distinguished based on molecular data. *Ceratomyxa cardinalis* (JX971431) and *C. cribbi* (EU440367) shared only 97.0% and 96.5% of similarity with *C. sargus* n. sp., respectively, with all others displaying similarity values lower than 90.0%. Distance estimation determined highest similarity to *Ceratomyxa* sp. SAR (DQ333430) (99.4%), *Ceratomyxa gunterae* (JX971422) (98.2%), and *Ceratomyxa archamiae* (JX971428) (98.1%). Other congeners having similar myxospores, but lacking molecular data allowing prompt differentiation, are C. *australis* Gaevskaya & Kovaleva, 1979, *C. arripica* Su & White, 1994, *C. castigatoides* Meglitsch, 1960, *C. declivis* Meglitsch, 1960, *C. simplex* Zhao et al., 2015, and *C. sprenti* Moser, Kent & Dennis, 1989. However, the myxospores of *C. australis* have smaller thickness range and thinner polar capsules (Eiras, 2006). *Ceratomyxa arripica* myxospores are narrower, with spherical polar capsules displaying a lower number of polar tubule coils (3–4) in a single row (Su & White, 1994). The myxospores of *C. castigatoides*, *C. declivis* and *C. simplex* have similar morphometry to those of *C. sargus* n. sp. but differ in terms of shape. *Ceratomyxa castigatoides* by having a posterior margin varying from convex to concave and spherical polar capsules, in addition to being slightly longer than the myxospores of *C. sargus* n. sp.; *C. declivis* by having a plump crescent shape with nearly truncated ends; and *C. simplex* by being strongly arcuate, with the anterior edge parallel to and almost symmetrical with the posterior edge, with both ends truncated (Eiras, 2006; Zhao et al., 2015). Finally, the myxospores of *C. sprenti* differ from those of *C. sargus* n. sp. by having both the anterior and posterior margins straight, with spherical polar capsules, and 5–6 coils of the polar tubule in a single row. They are also slightly thicker than the myxospores of *C. sargus* n. sp. (Moser, Kent & Dennis, 1989).

**Phylogenetic analysis**

**Figure 4:**
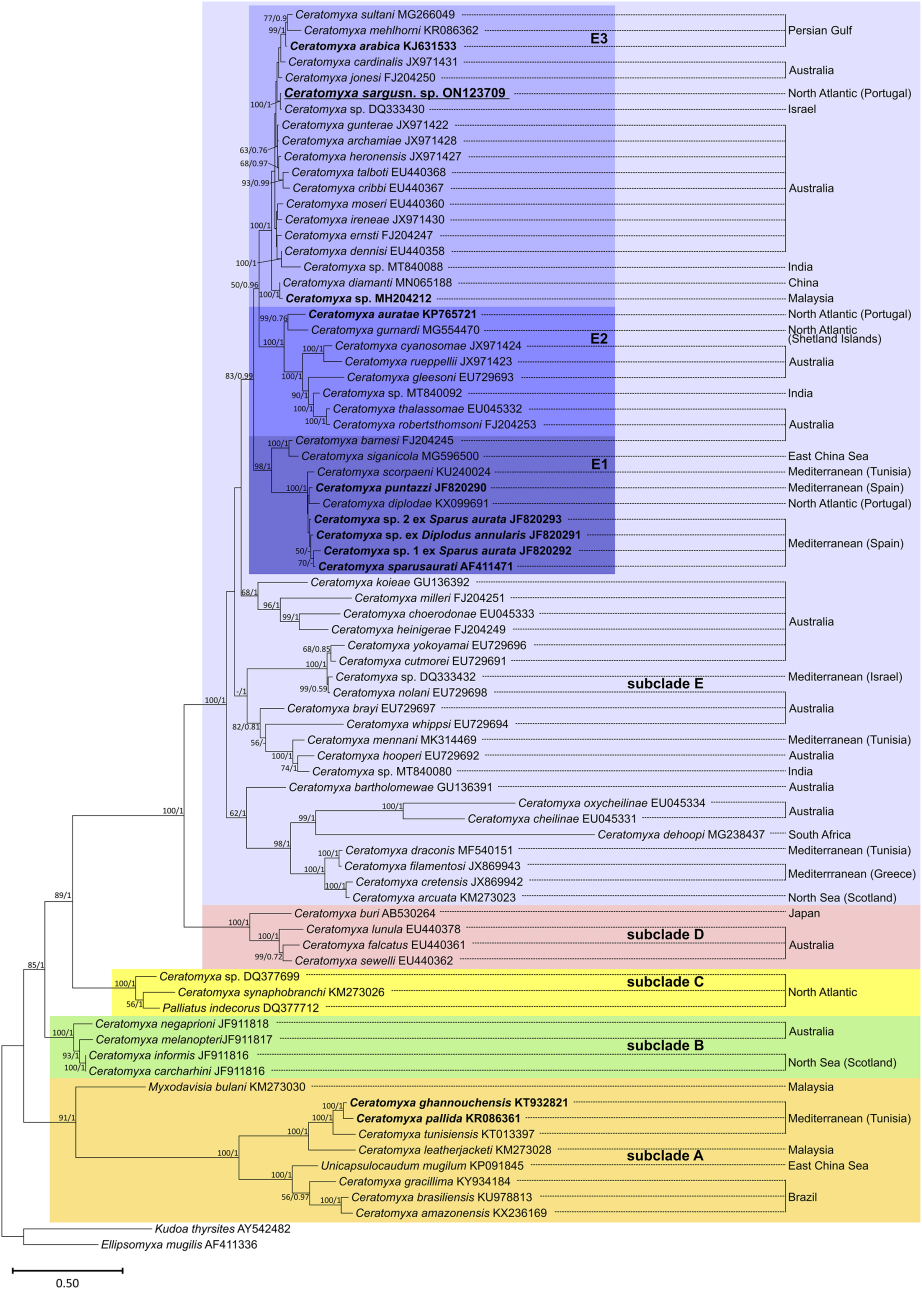
SSU rRNA-based maximum likelihood phylogenetic tree showing the position of sparid-infecting ceratomyxids (marked in bold) within the *Ceratomyxa* clade. Nodal supports are maximum likelihood bootstrap values and Bayesian inference probabilities; dashes represent poorly resolved nodes or a different branching pattern in the BI phylogenetic tree; nodes without values were poorly resolved in both trees.

Phylogenetic analysis based on SSU rRNA gene sequences showed the sequences available for sparid-infecting *Ceratomyxa*, including *C. sargus* n. sp., positioned within subclades A and E (Fig. 4). *Ceratomyxa pallida* (KR086361) and *C. ghannouchensis* (KT932821) from the sparid *Boops boops* in Tunisia cluster in the basal subclade A together with *C. tunisiensis* (KT013097) from Carangiformes also in Tunisia, and *C. leatherjacketi* (KM273028) from *Aluterus monoceros* (L.) (Tetraodontiformes) in Malaysia. The remaining sequences of sparid-infecting *Ceratomyxa* spp., including *C. sargus* n. sp., all clustered within subclade E, which is the most recent and taxon-rich subclade with unresolved deeper nodes. *Ceratomyxa sparusaurati* (AF411471), *Ceratomyxa* sp. 1 ex *S. aurata* (JF820292), *Ceratomyxa* sp. 2 ex *S. aurata* (JF820293), *C. puntazzi* (JF820290), and *Ceratomyxa* sp. ex *D. annularis* (JF820291) from South European *S. aurata* all cluster together to form the subclade E1, which further includes perciform-infecting species, namely *C. scorpaeni* (KU240024) from Tunisia, *C. siganicola* (MG596500) from the East China Sea, and *C. barnesi* (FJ204245) from Australia. The allegedly sparid-infecting species *Ceratomyxa diplodae* (KX099691) also clusters within subclade E1, despite its only available sequence having been obtained from infections in the gall bladder of European seabass *Dicentrarchus labrax* (Eupercaria *incertae sedis*). *Ceratomyxa auratae* (KP765721) from South European *S. aurata* clusters with *C. gurnardi* (MG554470) from the Atlantic Ocean off Scotland, in close relationship with several *Ceratomyxa* spp. from Kurtiformes and Perciformes in Australian waters, forming the subclade E2. Lastly, *C. sargus* n. sp. (ON123709) clusters within the subclade E3, which further comprises *C. arabica* (KJ631533) and a *Ceratomyxa* sp. (MH204212), also from sparid hosts in the Persian Gulf and Malaysia, respectively, plus an array of *Ceratomyxa* spp. mostly from Australian Pomacentridae (Ovalentaria *incertae sedis*) and Kurtiformes. Although the formation of these three subclades is well-supported in both maximum likelihood and Bayesian inference analyses, their exact relationship within subclade E remains uncertain. The inner topology of subclades E1 and E2 further display soft polytomies congruent with highly unresolved inner nodes.

***Zschokkella auratis* Rocha et al., 2013**

Fifteen out of the 54 specimens analysed (27.8%) from the fish farm located near the city of Portimão presented infection by this species, which was co-infective with *C. sargus* n. sp. in four specimens. Infections by *Z. auratis* were not detected in samples obtained from the EPPO. Species identification was based on the morphological and morphometric aspects of the myxospores, and on molecular data of the SSU rRNA gene.

**Figure 5:**
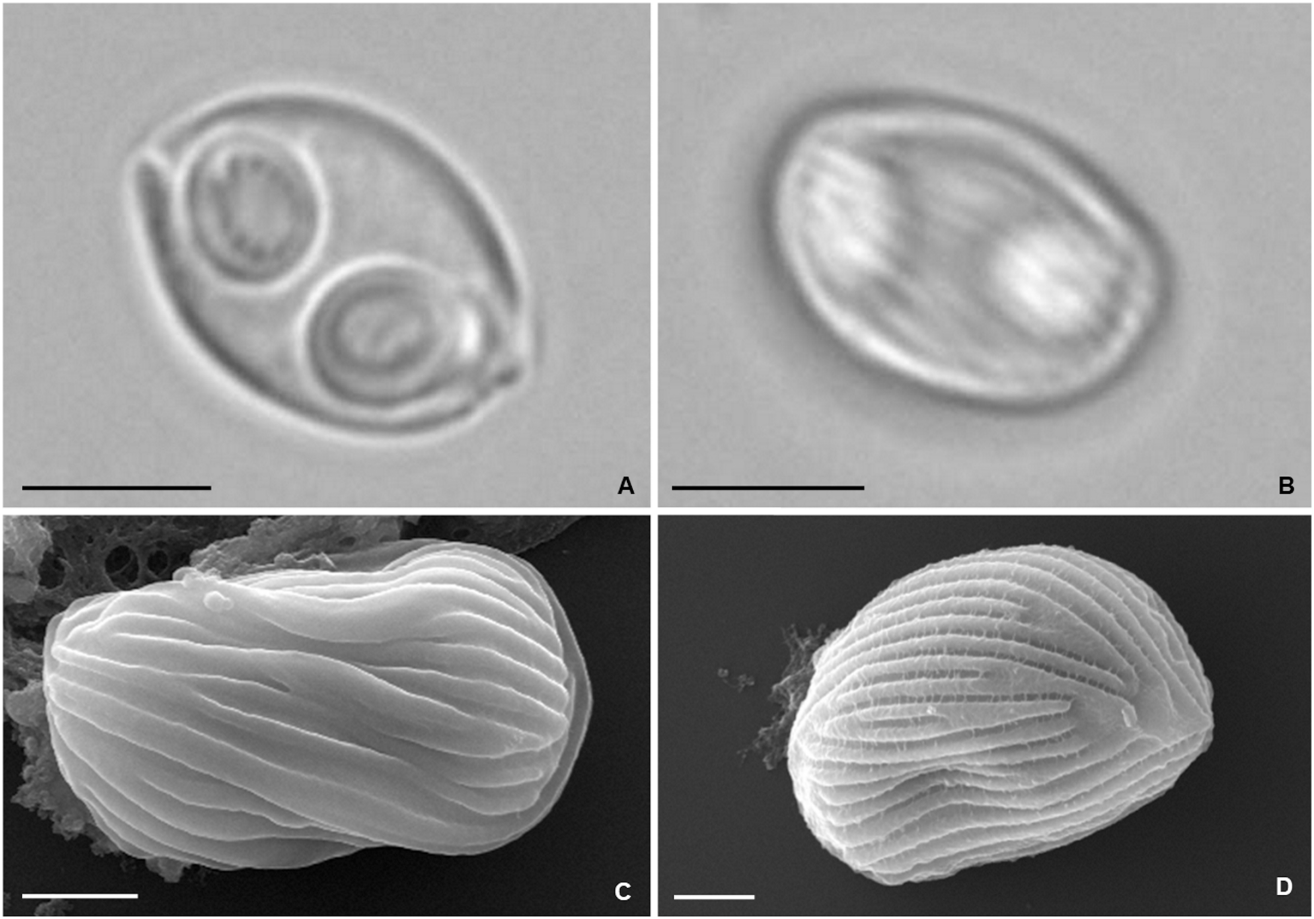
Photomicrographs of *Zschokkella auratis* from the gall bladder of *Diplodus sargus*. (A) Light micrograph showing a myxospore ovoidal in sutural view, and containing two subspherical polar capsules located subterminally and opening at nearly opposite positions. Scale bar = 5 µm. (B) Light micrograph of a myxospore evidencing the presence of surface ridges. Scale bar = 5 µm. (C) SEM micrograph of a myxospore in sutural view, showing the surface ridges organized parallel to the suture line. Scale bar = 2 µm. (D) SEM micrograph allowing recognition of the surface pattern formed by the ridges that extend throughout the myxospore body. Scale bar = 2 µm.

Mature myxospores observed isolated in the bile were ovoidal in sutural and valvular views, with rounded opposite sides, measuring 9.6 ± 0.5 (9.1−10.3) μm in length and 6.7 ± 0.2 (6.4−7.0) μm in width. Myxospore wall thick, composed of two symmetrical valves united along a curved suture line (Figs. 5A and 5B). Light microscopy and SEM observations revealed the presence of numerous surface ridges organized parallel to the suture line and forming a pattern along the entire myxospore body (Figs. 5B–5D). Two symmetrical subspherical polar capsules, 3.7 ± 0.4 (3.0−4.2) μm long and 3.0 ± 0.2 (2.7−3.3) μm wide, were located sub terminally at the same level within the myxospores and opened at nearly opposite positions. Each polar capsule contained a polar tubule coiled in four to five turns (Fig. 5A). These morphological and morphometric features are largely congruent with those reported in the original description of *Z. auratis* (Rocha et al., 2013).

The partial SSU rRNA gene sequence obtained from the isolate comprised 2,055 bp and is deposited in GenBank under the accession no. ON054249. Distance estimation confirmed species identification, having revealed 99.6% similarity to the sequence of *Z. auratis* (KC849425) available from gallbladder infections in specimens of *S. aurata* also reared in Southern Portuguese fish farms (Rocha et al., 2013), and 99.0% to a second sequence of *Z. auratis* (MF978273) obtained from the brain of farmed striped snakehead *Channa striata* (Bloch, 1793) in India (Paul et al., 2020). All other sequences retrieved less than 94.5% similarity.

## Discussion

The present study reports *Ceratomyxa sargus* n. sp. and *Zschokkella auratis* Rocha et al., 2013 infecting the gallbladder of reared white seabream *D. sargus* from two Southern Portuguese fish farms. While *C. sargus* n. sp. was present at both sampling locations, *Z. auratis* was never observed in specimens obtained from the EPPO. We suspect this to be correlated with differences in the annelid communities established in the earth ponds of the two fish farms and nearby wild locations in the affluents used for water supply. In order to evaluate this hypothesis and understand if infections of reared specimens are dependent or not on the earth pond annelids, we are presently surveying the annelid communities present at these locations for the detection of actinospore development.

The species identifications performed in this study were based on combined microscopic and molecular data. Ultrastructure was further performed for *C. sargus* n. sp. and revealed developmental features congruent with previous studies of *Ceratomyxa*, *i.e*., asynchronous development, with pseudoplasmodia displaying different shapes and sizes, and lacking the peripheral projections typical of other myxosporean coelozoic plasmodia (Rocha et al., 2015, 2016). The coiling of the polar tubule in two rows, although uncommon among Myxosporea, has been reported in ultrastructural studies of other *Ceratomyxa* spp., such as *C. diplodae* (Rocha et al., 2016) and *Ceratomyxa tenuispora* (Casal, Costa & Azevedo, 2007).

Molecular comparison to myxozoan sequences available in GenBank confirmed the identity of the *Zschokkella* samples here studied to *Z. auratis* (KC849425) with 99.6% of similarity, and revealed *C. sargus* n. sp. presenting 99.4% similarity to the sequence of an undescribed *Ceratomyxa* sp. (DQ333430). Though genetic differences higher than 1% are usually accepted for establishing myxozoan differentiation, there is no specified benchmark for *Ceratomyxa* interspecific variation, making it necessary for species identifications to be corroborated by differences in other taxonomic characters such as myxospore morphometry, host species, and tissue specificity (Gunter, Whipps & Adlard, 2009; Heiniger & Adlard, 2013; Atkinson et al., 2015; Bartošová-Sojková et al., 2018). According to the GenBank record, *Ceratomyxa* sp. (DQ333430) was sequenced from the gall bladder of marbled spinefoot *Siganus rivulatus* Forsskål & Niebuhr, 1775 (Acanthuriformes, Siganidae) from Israel. No data are available for myxospore morphology and dimensions. Thus, undoubtable conspecificity between these two isolates cannot be solely based on the small genetic difference found between their sequences (0.6%). Future analyses targeting the re-sequencing and morphological study of the Israeli isolate are necessary to either confirm or deny conspecificity to *C. sargus* n. sp., and acknowledge host specificity of this species.

Although ceratomyxids are usually accepted to be host specific (Gunter & Adlard, 2009; Gunter, Whipps & Adlard, 2009; Heiniger & Adlard, 2013; Alama-Bermejo, Raga & Holzer, 2011), there are molecularly confirmed exceptions to this rule (see Gunter & Adlard, 2008; Heiniger & Adlard, 2013; Fiala et al., 2015; Thabet et al., 2016; Bartošová-Sojková et al., 2018). Among sparid-infecting *Ceratomyxa* spp., *Ceratomyxa diplodae* Lubat et al., 1989 and *C. pallida* have been reported from multiple hosts but were characterized based on myxospore morphology alone. *Ceratomyxa diplodae* was originally described from the gall bladder of the annular seabream *Diplodus annularis*, and later reported from *D. labrax*, *D. dentex* and *D. puntazzo* (Lubat et al., 1989; Alvarez-Pellitero & Sitjà-Bobadilla, 1993; Rigos et al., 1997; Katharios et al., 2007), while *C. pallida* has been reported to infect both *S. salpa* and *B. boops* (Thélohan, 1895; Thabet et al., 2019). Considering that morphology alone is also a weak character for determining conspecificity among Myxosporea (Atkinson et al., 2015), molecular data are needed from these species to better support hypotheses and speculation about host specificity and potential host shifting/switching of sparid-infecting *Ceratomyxa*.

The phylogenetic analysis performed here is congruent with previous studies through supporting a close phylogenetic relationship between *Ceratomyxa* spp. The SSU rRNA gene sequences belonging to this genus have been shown to cluster together, distributed among five well-defined subclades that further encompass representatives of the genera *Palliatus*, *Pseudoalataspora*, *Myxodavisia* and *Unicapsulocaudum* (Fiala et al., 2015; Rocha et al., 2015; Yang et al., 2017). This topology was retrieved in our phylogenetic trees, which showed subclade E as the most divergent and taxon-rich subclade displaying unresolved deeper nodes, in accordance with previous studies (Gunter, Whipps & Adlard, 2009; Fiala et al., 2015; Bartošová-Sojková et al., 2018).

Overall, the sequences available for sparid-infecting *Ceratomyxa*, including *C. sargus* n. sp., were retrieved positioned within subclades A, E1, E2, and E3, forming several independent lineages of sparid-infecting *Ceratomyxa* spp. Their radiation within the most divergent subclades (E1–E3) further supports the occurrence of more recent evolutionary events driving rapid speciation of these parasites in sparid fish. This is suggested by the soft inner polytomies in subclades E1 and E3, which demonstrate rapid species evolution, with the data currently available being unable to resolve inner nodes and ascertain exact interspecific relationships within these groups.

The sequence obtained for *C. sargus* n. sp. appeared positioned separately from other sparid-infecting *Ceratomyxa* spp. reported from Southern European countries, demonstrating that this species does not share a more immediate common ancestor with its closest relatives based on host affinity and geography. This reinforces the occurrence of multiple evolutionary entries of *Ceratomyxa* into sparid fish in the Southern European region alone. Our study further strengthens the contention that geographic location is not a main driver of *Ceratomyxa* radiation (Gunter & Adlard, 2009; Alama-Bermejo, Raga & Holzer, 2011; Rocha et al., 2015), as it shows *C. sargus* n. sp. clustering together with *Ceratomyxa* spp. from the Persian Gulf and Australia, rather than with other Southern European species.

Molecular systematics have shown a surprising radiation of *Ceratomyxa* within certain host families (Heiniger, Gunter & Adlard, 2008; Gunter & Adlard, 2009; Alama-Bermejo, Raga & Holzer, 2011; Heiniger & Adlard, 2013; Bartošová-Sojková et al., 2018). *Ceratomyxa sargus* n. sp. is the 12^th^ species of the genus described from sparid fishes in the Mediterranean and North Atlantic off Southern European regions. This supports the existence of a high species richness in sparids inhabiting this geographic region, demonstrating the need for integrative taxonomic studies to shed insight into this host-parasite association with potential economic impact for aquaculture and fishery industries. Pathological changes induced by *Ceratomyxa* are mostly mild and may include swelling, vacuolization, sloughing, damage, and necrosis of the gallbladder’ epithelial cells (Sitjà-Bobadilla & Alvarez-Pellitero, 1993; Palenzuela, Sitjà-Bobadilla & Alvarez-Pellitero, 1997), but high mortality rates have also been associated with these myxosporeans (Katharios et al., 2007). Despite clinical signs of infection and disease having not been observed in *D. sargus* specimens infected with *C. sargus* n. sp. and *Z. auratis*, it is our intention to proceed with pathological studies trying to assess the real pathogenicity and impact that these parasites might have on cultured stocks.

Our study reports for the first time the occurrence of *Zschokkella auratis* in *D. sargus*. This myxosporean was originally described from the gall bladder of another sparid fish, the gilthead seabream *S. aurata*, also reared in a Southern Portuguese fish farm (Rocha et al., 2013). A second report was recently performed from infections in the brain of farmed striped snakehead *Channa striata* (Anabantiformes, Channidae) in India (Paul et al., 2020). However, species identification was based in a short 618 bp-long SSU rRNA fragment (MF978273) at a highly conservative region close to the 3′ end of the gene and cannot provide undoubtable evidence of conspecificity.

The occurrence of *Z. auratis* in more than a single host species agrees with the broad host specificity that has been reported for *Zschokkella*. According to a recent synopsis, 24 *Zschokkella* spp. have been reported from multiple hosts; half from various fishes belonging to the same taxonomic order, even if from distinct families, and the other half from a broader range of taxonomically distant hosts (Matsche et al., 2021). For instance, *Z. leptatherinae* Su & White, 1995 and *Z. macrocapsula* Su & White, 1995 have been reported from multiple Atheriniformes; *Z. hildae* Auerbach, 1910, *Z. meglitschi* Moser & Noble, 1977 and *Z. microcapsula* Moser & Noble, 1977 from multiple Gadiformes; and *Z. carassii* Nie & Li, 1973, *Z. oviformis* Ma et al., 1982 and *Z. striata* Shulman, 1962 from multiple Cypriniformes. In turn, *Z. nova* Klokačewa, 1914 has been reported from an array of fish species belonging to the orders Cypriniformes, Salmoniformes, Perciformes, Mugiliformes and Anguiliformes; *Z. candia* Kalatzis et al., 2015 from fishes belonging to Perciformes, Cichliformes and Eupercaria *incertae sedis*; with several others reported from fish species belonging to at least two taxonomic orders (Matsche et al., 2021). Most of these reports were performed based solely on myxospore morphology, for which this broad host specificity requires corroboration through sequencing of species isolates from supposed hosts.

The molecular data available for the genus *Zschokkella* is rather limited, representing ca. 15% of the number of species thus far described, and including information for different host isolates of only *Z. candia*, *Z. nova*, and now also *Z. auratis*. Identical SSU rRNA gene sequences are available in GenBank for *Z. candia* infections in *Sparisoma cretense* (Eupercaria *incertae sedis*), *Scorpaena porcus* (Perciformes), and *Oreochromis niloticus* (Cichliformes). *Zschokkella nova* accounts for a total of 10 sequences from the SSU and LSU genetic markers ascertaining identification of the parasite in *Carassius auratus gibelio*, *Ctenopharyngodon idella* (Cypriniformes), and *Sander lucioperca* (Perciformes). The present study further reveals molecular evidence for the development of *Z. auratis* in at least two sparid fish, showcasing a potential host shift phenomenon of this myxozoan among sparids. This highlights a necessity for myxozoans surveys to target the molecular description of known *Zschokkella* spp., to determine their true host range and capacity of this morphotype to undertake host shift/witch.

## Conclusions

The present study sheds some insight into the diversity of myxosporean parasites potentially affecting the sustainable production of white seabream *Diplodus sargus* in Southern European aquacultures. Two gall bladder dwelling myxosporeans are reported from specimens reared in Southern Portuguese fish farms. The description of *Ceratomyxa sargus* n. sp. reinforces the previously reported high species richness of *Ceratomyxa* infecting sparids in the Southern European region, with phylogenetic analyses demonstrating the lack of a more immediate common ancestor for these species and highlighting multiple evolutionary entries into this host family in Southern Europe. The possibility of conspecificity between *C. sargus* n. sp. and the unnamed *Ceratomyxa* sp. reported from the acanthuriform *Siganus rivulatus* in Israel requires investigation through the morphological and molecular analysis of novel material from the Israeli isolate, as confirmation of conspecificity would provide evidence of host switch potentially allowing geographical spreading of *C. sargus* n. sp. from co-habitation sites in the eastern Mediterranean.

Contextualization of evidence of host shift of *Zschokkella auratis* among sparids with the available literature suggest a high capability of the *Zschokkella* morphotype to undergo host shift/switch that requires confirmation through the molecular description of the numerous *Zschokkella* spp. reported from multiple hosts and geographical locations.

## Supplemental Information

10.7717/peerj.14599/supp-1 Supplemental Information 1 SSU rRNA sequence alignment used for the phylogenetic analysis of Ceratomyxa sargus n. sp. and closest relatives based on host affinity and geographic location.

## Additional Information and Declarations

### DNA Deposition

The following information was supplied regarding the deposition of DNA sequences: The novel SSU rRNA gene sequence of Zschokkella auratis is available at GenBank: ON054249. The SSU rRNA gene sequence of Ceratomyxa sargus n. sp. is available at GenBank: ON123709.

### Data Availability

The following information was supplied regarding data availability: The raw data is available in the Supplemental File.

### New Species Registration

The following information was supplied regarding the registration of a newly described species: Ceratomyxa sargus species LSID: urn:lsid:zoobank.org:act:3103EB6E-000F-445C-AA10-F129C0509EFC. Publication LSID: urn:lsid:zoobank.org:pub:E70B1814-0651-4396-81B4-0CFECEC525E2.

## References

[ref-1] Alama-Bermejo G, Hernandez-Orts JS, Huchon D, Atkinson SD (2021). Two novel myxosporean parasite species of *Ceratomyxa* Thélohan, 1892 from the banded cusk-eel *Raneya brasiliensis* (Kaup) (Ophidiiformes: Ophidiidae) off Patagonia, Argentina. Parasitology International.

[ref-2] Alama-Bermejo G, Raga JA, Holzer AS (2011). Host-parasite relationship of *Ceratomyxa puntazzi* n. sp. (Myxozoa: Myxosporea) and sharpsnout seabream *Diplodus puntazzo* (Walbaum, 1792) from the Mediterranean with first data on ceratomyxid host specificity in sparids. Veterinary Parasitology.

[ref-3] Alvarez-Pellitero P, Sitjà-Bobadilla A (1993). *Ceratomyxa* spp. (Protozoa: Myxosporea) infections in wild and cultured sea bass, *Dicentrarchus labrax*, from the Spanish Mediterranean area. Journal of Fish Biology.

[ref-4] Athanassopoulou F, Prapas T, Rodger H (1999). Diseases of *Puntazzo puntazzo* Cuvier in marine aquaculture systems in Greece. Journal of Fish Diseases.

[ref-5] Atkinson SD, Bartošová-Sojková P, Whipps CM, Bartholomew JL, Okamura B, Gruhl A, Bartholomew JL (2015). Approaches for characterising myxozoan species. Myxozoan Evolution, Ecology and Development.

[ref-6] Bartošová P, Fiala I, Hypša V (2009). Concatenated SSU and LSU rDNA data confirm the main evolutionary trends within myxosporeans (Myxozoa: Myxosporea) and provide an effective tool for their molecular phylogenetics. Molecular Phylogenetics and Evolution.

[ref-7] Bartošová-Sojková P, Lövy A, Reed CC, Lisnerová M, Tomková T, Holzer AS, Fiala I (2018). Life in a rock pool: radiation and population genetics of myxozoan parasites in hosts inhabiting restricted spaces. PLOS ONE.

[ref-8] Caffara M, Marcer F, Florio D, Quaglio F, Fioravanti ML (2003). Heart infection due to *Henneguya* sp (Myxozoa, Myxosporea) in gilthead sea bream (*Sparus aurata*) cultured in Italy. Bulletin of the European Association of Fish Pathologists.

[ref-9] Casal G, Costa G, Azevedo C (2007). Ultrastructural description of *Ceratomyxa tenuispora* (Myxozoa), a parasite of the marine fish *Aphanopus carbo* (Trichiuridae), from the Atlantic coast of Madeira Island (Portugal). Folia Parasitol.

[ref-10] Castresana J (2000). Selection of conserved blocks from multiple alignments for their use in phylogenetic analysis. Molecular Biology and Evolution.

[ref-11] Costa G, Lom J, Andrade C, Barradas R (1998). First report of *Ceratomyxa sparusaurati* (Protozoa: Myxosporea) and the occurrence of epitheliocystis in cultured sea bream, *Sparus aurata* L. from Madeira. Bulletin of the European Association of Fish Pathologists.

[ref-12] Dereeper A, Guignon V, Blanc G, Audic S, Buffet S, Chevenet F, Dufayard JF, Guindon S, Lefort V, Lescot M, Claverie JM, Gascuel O (2008). Phylogeny.fr: robust phylogenetic analysis for the non-specialist. Nucleic Acids Research.

[ref-13] Diamant A, Lom J, Dyková I (1994). *Myxidium leei* n. sp., a pathogenic myxosporean of cultured sea bream *Sparus aurata*. Diseases of Aquatic Organisms.

[ref-14] Diamant A, Ucko M, Paperna I, Colorni A, Lipshitz A (2005). *Kudoa iwatai* (Myxosporea: Multivalvulida) in wild and cultured fish in the Red Sea: redescription and molecular phylogeny. Journal of Parasitology.

[ref-15] Eiras JC (2006). Synopsis of the species of *Ceratomyxa* Thélohan, 1892 (Myxozoa: Myxosporea: Ceratomyxidae). Systematic Parasitology.

[ref-16] Eiras JC, Cruz C, Saraiva A (2018). Synopsis of the species of *Ceratomyxa* Thélohan, 1892 (Cnidaria, Myxosporea, Ceratomyxidae) described between 2007 and 2017. Systematic Parasitology.

[ref-17] Fiala I (2006). The phylogeny of Myxosporea (Myxozoa) based on small subunit ribosomal RNA gene analysis. International Journal for Parasitology.

[ref-18] Fiala I, Hlavničková M, Kodádková A, Freeman MA, Bartošová-Sojková P, Atkinson SD (2015). Evolutionary origin of *Ceratonova shasta* and phylogeny of the marine myxosporean lineage. Molecular Phylogenetics and Evolution.

[ref-19] Georgévitch J (1916). Note sur les myxosporidies des poissons de la baie de Vilefranche et de Monaco. Bulletin de l’Institut océanographique de Monaco.

[ref-20] Golomazou E, Athanasopoulou F, Vagianou S, Sabatakou O, Tsantilas I, Kokokiris L (2006). Diseases of white sea bream (*Diplodus sargus* L.) reared in experimental and commercial conditions in Greece. Turkish Journal of Veterinary and Animal Sciences.

[ref-21] Golomazou E, Athanassopoulou F, Karagouni E, Kokkokiris L (2009). The effect of seasonality on the health and growth of a newly recorded *Myxobolus* species infecting cultured sharp snout seabream (*Diplodus puntazzo* C.). Turkish Journal of Veterinary and Animal Sciences.

[ref-22] Gunter NL, Adlard RD (2008). Bivalvulidan (Myxozoa: Myxosporea) parasites of damselfishes with description of twelve novel species from Australia’s Great Barrier Reef. Parasitology.

[ref-23] Gunter NL, Adlard RD (2009). Seven new species of *Ceratomyxa* Thelohán, 1892 (Myxozoa) from the gall-bladders of serranid fishes from the Great Barrier Reef, Australia. Systematic Parasitology.

[ref-24] Gunter NL, Whipps CM, Adlard RD (2009). *Ceratomyxa* (Myxozoa: Bivalvulida): robust taxon or genus of convenience?. International Journal for Parasitology.

[ref-25] Hallett SL, Diamant A (2001). Ultrastructure and small-subunit ribosomal DNA sequence of *Henneguya lesteri* n. sp. (Myxosporea), a parasite of sand whiting *Sillago analis* (Sillaginidae) from the coast of Queensland, Australia. Diseases of Aquatic Organisms.

[ref-26] Heiniger H, Adlard RD (2013). Molecular identification of cryptic species of *Ceratomyxa* Thelohán, 1892 (Myxosporea: Bivalvulida) including the description of eight novel species from apogonid fishes (Perciformes: Apogonidae) from Australian waters. Acta Parasitologica.

[ref-27] Heiniger H, Gunter NL, Adlard RD (2008). Relationships between four novel ceratomyxid parasites from the gall bladders of labrid fishes from Heron Island, Queensland, Australia. Parasitology International.

[ref-28] Hillis DM, Dixon MT (1991). Ribosomal DNA: molecular evolution and phylogenetic inference. The Quarterly Review of Biology.

[ref-29] Holzer AS, Sommerville C, Wootten R (2004). Molecular relationships and phylogeny in a community of myxosporeans and actinosporeans based on their 18S rDNA sequences. International Journal for Parasitology.

[ref-30] Kalavati C, MacKenzie K (1999). The genera *Ceratomyxa* Thélohan, 1892, *Leptotheca* Thélohan, 1895 and *Sphaeromyxa* Thélohan, 1892 (Myxosporea: Bivalvulida) in gadid fish of the northeast Atlantic. Systematic Parasitology.

[ref-31] Katharios P, Garaffo M, Sarter K, Athanassopoulou F, Mylonas CC (2007). A case of high mortality due to heavy infestation of *Ceratomyxa diplodae* in sharpsnout sea bream (*Diplodus puntazzo*) treated with reproductive steroids. Bulletin of the European Association of Fish Pathologists.

[ref-32] Kent ML, Andree KB, Bartholomew JL, El-Matbouli M, Desser SS, Devlin RH, Feist SW, Hedrick RP, Hoffmann RW, Khattra J, Hallett SL, Lester RJ, Longshaw M, Palenzeula O, Siddall ME, Xiao C (2001). Recent advances in our knowledge of the Myxozoa. The Journal of Eukaryotic Microbiology.

[ref-33] Kumar S, Stecher G, Li M, Knyaz C, Tamura K (2018). MEGA X: molecular evolutionary genetics analysis across computing platforms. Molecular Biology and Evolution.

[ref-34] Lom J, Arthur JR (1989). A guideline for the preparation of species descriptions in Myxosporea. Journal of Fish Diseases.

[ref-35] Lom J, Dyková I (2006). Myxozoan genera: definition and notes on taxonomy, life-cycle terminology and pathogenic species. Folia Parasitologica.

[ref-36] Lubat J, Radujkovic B, Marques A, Bouix G (1989). Parasites des poissons marins du Montenegro: Myxosporidies. Acta Adriatica.

[ref-37] Matsche MA, Yurakhno V, Zhang J, Sato H (2021). Synopsis of the species of the genus *Zschokkella* Auerbach, 1910 (Myxozoa: Bivalvulida: Myxidiidae). Systematic Parasitology.

[ref-38] Moser M, Kent M, Dennis D (1989). Gall-bladder Myxosporea in coral-reef fishes from Heron Island, Australia. Australian Journal of Zoology.

[ref-39] Nei M, Kumar S (2000). Molecular evolution and phylogenetics.

[ref-40] Okamura B, Gruhl A, Bartholomew JL, Okamura B, Gruhl A, Bartholomew JL (2015). An introduction to myxozoan evolution, ecology and development. Myxozoan Evolution, Ecology and Development.

[ref-41] Palenzuela O, Sitjà-Bobadilla A, Alvarez-Pellitero P (1997). *Ceratomyxa sparusaurati* (Protozoa: Myxosporea) infections in cultured gilthead sea bream *Sparus aurata* (Pisces: Teleostei) from Spain: aspects of the host parasite relationship. Parasitology Research.

[ref-42] Paul A, Pattanayak S, Sahoo MK, Kumar PR, Kumar R, Sahoo PK (2020). Co-infection of bacterial and parasitic pathogens including the myxosporean *Zschokkella auratis* infecting brain in farmed striped murrel *Channa striata* (Bloch, 1793) causing large scale mortality. Indian Journal of Fisheries.

[ref-43] Rangel LF, Castro R, Rocha S, Severino R, Casal G, Azevedo C, Cavaleiro F, Santos MJ (2016). Tetractinomyxon stages genetically consistent with *Sphaerospora dicentrarchi* (Myxozoa: Sphaerosporidae) found in *Capitella* sp. (Polychaeta: Capitellidae) suggest potential role of marine polychaetes in parasite’s life cycle. Parasitology.

[ref-44] Rangel LF, Rocha S, Borkhanuddin MH, Cech G, Castro R, Casal G, Azevedo C, Severino R, Székely C, Santos MJ (2014). *Ortholinea auratae* n. sp. (Myxozoa, Ortholineidae) infecting the urinary bladder of the gilthead seabream *Sparus aurata* (Teleostei, Sparidae), in a Portuguese fish farm. Parasitology Research.

[ref-45] Rangel LF, Rocha S, Casal G, Castro R, Severino R, Azevedo C, Cavaleiro F, Santos MJ (2017). Life cycle inference and phylogeny of *Ortholinea labracis* n. sp. (Myxosporea: Ortholineidae), a parasite of the European seabass *Dicentrarchus labrax* (Teleostei: Moronidae), in a Portuguese fish farm. Journal of Fish Diseases.

[ref-46] Rigos G, Grigorakis K, Christophilogiannis P, Nengas I, Alexis M (1997). *Ceratomyxa* spp. (Myxosporea) infection in cultured common dentex from Greece. Bulletin of the European Association of Fish Pathologists.

[ref-47] Rocha S, Casal G, Rangel L, Castro R, Severino R, Azevedo C, Santos MJ (2015). Ultrastructure and phylogeny of *Ceratomyxa auratae* n. sp. (Myxosporea: Ceratomyxidae), a parasite infecting the gilthead seabream *Sparus aurata* (Teleostei: Sparidae). Parasitology International.

[ref-48] Rocha S, Casal G, Rangel L, Severino R, Castro R, Azevedo C, Santos MJ (2013). Ultrastructural and phylogenetic description of *Zschokkella auratis* sp. nov. (Myxozoa), a parasite of the gilthead seabream *Sparus aurata*. Diseases of Aquatic Organisms.

[ref-49] Rocha S, Rangel LF, Castro R, Severino R, Azevedo C, Santos MJ, Casal G (2016). Ultrastructure and phylogeny of *Ceratomyxa diplodae* (Myxosporea: Ceratomyxidae), from gall bladder of European seabass *Dicentrarchus labrax*. Diseases of Aquatic Organisms.

[ref-50] Ronquist F, Huelsenbeck JP (2003). MrBayes 3: Bayesian phylogenetic inference under mixed models. Bioinformatics.

[ref-51] Santos MJ (1996). Observations on the parasitofauna of wild sea bass (*Dicentrarchus labrax* L.) from Portugal. Bulletin of the European Association of Fish Pathologists.

[ref-52] Sirin C, Santos MJ, Rangel LF (2018). Morphological and molecular analyses of *Bipteria lusitanica* n. sp. in wild white seabream, *Diplodus sargus* (Linnaeus, 1758) in Portugal. Parasitology Research.

[ref-53] Sitjà-Bobadilla A, Alvarez-Pellitero P (1993). Light and electron microscopical description of *Ceratomyxa labracis* n. sp. and a redescription of *C. diplodae* (Myxosporea: Bivalvulida) from wild and cultured Mediterranean sea bass *Dicentrarchus labrax* (L.) (Teleostei: Serranidae). Systematic Parasitology.

[ref-54] Sitjà-Bobadilla A, Alvarez-Pellitero P (1995). Light and electron microscopic description of *Polysporoplasma* n. g. (Myxosporea: Bivalvulida), *Polysporoplasma sparis* n. sp. from *Sparus aurata* (L), and *Polysporoplasma mugilis* n. sp. from *Liza aurata* L. European Journal of Protistology.

[ref-55] Sitjà-Bobadilla A, Alvarez-Pellitero P (2001). *Leptotheca sparidarum* n. sp. (Myxosporea: Bivalvulida), a parasite from cultured common dentex (*Dentex dentex* L.) and gilthead sea bream (*Sparus aurata* L.) (Teleostei: Sparidae). Journal of Eukaryotic Microbiology.

[ref-56] Sitjà-Bobadilla A, Palenzuela O, Álvarez-Pellitero P (1995). *Ceratomyxa sparusaurati* n. sp. (Myxosporea, Bivalvulida), a new parasite from cultured gilthead seabream (*Sparus aurata* L.) (Teleostei, Sparidae)—light and electron microscopic description. The Journal of Eukaryotic Microbiology.

[ref-57] Su XQ, White RWG (1994). New myxosporeans (Myxozoa, Myxosporea) from marine fishes of Tasmania, Australia. Acta Protozoologica.

[ref-59] Thabet A, Abdel-Baki AAS, Harrath AH, Mansour L (2019). Morphological and molecular aspects of *Ceratomyxa ghannouchensis* n. sp. and *C. pallida* Thelohán 1894 infecting the bogue, *Boops boops* (l.). Journal of Natural History.

[ref-60] Thabet A, Mansour L, Al Omar SY, Tlig-Zouari S (2016). *Ceratomyxa tunisiensis* n. sp. (Myxosporea: Bivalvulida) from the gallbladders of two carangid fish caught off the coast of Tunisia. Journal of Eukaryotic Microbiology.

[ref-61] Thélohan P (1895). Recherches sur les Myxosporidies. Bulletin Scientifique de la France et de la Belgique.

[ref-62] Whipps CM, Adlard RD, Bryant MS, Lester RJ, Findlay V, Kent ML (2003). First report of three *Kudoa* species from eastern Australia: *Kudoa thyrsites* from mahi mahi (*Coryphaena hippurus*), *Kudoa amamiensis* and *Kudoa minithyrsites* n. sp. from sweeper (*Pempheris ypsilychnus*). The Journal of Eukaryotic Microbiology.

[ref-63] Yang C, Zhou Y, Zhao Y, Huang W, Huang C (2017). Erection of *Unicapsulocaudum mugilum* gen. et sp. nov. (Myxozoa: Ceratomyxidae) based on its morphological and molecular data. Journal of Natural History.

[ref-64] Zhao Y, Al-Farraj SA, Al-Rasheid KAS, Song W (2015). Data on ten new myxosporean parasites (Myxozoa, Myxosporea, Bivalvulida) from the Yellow Sea, China. Acta Protozoologica.

